# 3D‐Printed Sugar Scaffold for High‐Precision and Highly Sensitive Active and Passive Wearable Sensors

**DOI:** 10.1002/advs.201902521

**Published:** 2019-11-11

**Authors:** Dong Hae Ho, Panuk Hong, Joong Tark Han, Sang‐Youn Kim, S. Joon Kwon, Jeong Ho Cho

**Affiliations:** ^1^ SKKU Advanced Institute of Nanotechnology (SAINT) Sungkyunkwan University (SKKU) Suwon 16419 Republic of Korea; ^2^ Nano Hybrid Technology Research Center Korea Electrotechnology Research Institute (KERI) Changwon 642‐120 Republic of Korea; ^3^ Interaction Laboratory Advanced Research Technology Center Computer Science and Engineering Korea University of Technology and Education Cheonan 330‐708 Korea; ^4^ Nanophotonics Research Center Korea Institute of Science and Technology (KIST) Seoul 02792 Republic of Korea; ^5^ Department of Chemical and Biomolecular Engineering Yonsei University Seoul 03722 Republic of Korea

**Keywords:** 3D printing, biosensors, lightweight, porous geometry, sugar scaffolds

## Abstract

In this study, a pairing of a previously unidentified 3D printing technique and soft materials is introduced in order to achieve not only high‐resolution printed features and flexibility of the 3D‐printed materials, but also its light‐weight and electrical conductivity. Using the developed technique and materials, high‐precision and highly sensitive patient‐specific wearable active or passive devices are fabricated for personalized health monitoring. The fabricated biosensors show low density and substantial flexibility because of 3D microcellular network‐type interconnected conductive materials that are readily printed using an inkjet head. Using high‐resolution 3D scanned body‐shape data, on‐demand personalized wearable sensors made of the 3D‐printed soft and conductive materials are fabricated. These sensors successfully detect both actively changing body strain signals and passively changing signals such as electromyography (EMG), electrodermal activity (EDA), and electroencephalogram EEG. The accurately tailored subject‐specific shape of the developed sensors exhibits higher sensitivity and faster real‐time sensing performances in the monitoring of rapidly changing human body signals. The newly developed 3D printing technique and materials can be widely applied to various types of wearable, flexible, and light‐weight biosensors for use in a variety of inexpensive on‐demand and personalized point‐of‐care diagnostics.

## Graphical abstract

A pairing of a 3D‐printing technique and soft materials is introduced to achieve not only high‐resolution printed features and flexibility of the 3D‐printed materials but also its lightweight and electrical conductivity. Using the developed technique and materials, high‐precision and highly sensitive patient‐specific wearable devices are fabricated for personalized health monitoring.

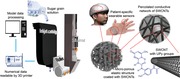

The rapid growth in demand for personalized medicine has created a proportional requirement for practical, inexpensive, and reliable devices and materials for point‐of‐care diagnostics.^1^, ^2^, ^3^ 3D printing has been actively employed to address these requirements, because it offers beneficial advantages such as cost‐effectiveness, portability, high resolution for printed features, and usability of a wide range of printable materials^4^ (i.e., metal,^5^, ^6^, ^7^, ^8^, ^9^ ceramics,^10^, ^11^, ^12^, ^13^, ^14^, ^15^ plastics,^16^, ^17^, ^18^, ^19^ and etc.). Indeed, leading healthcare providers have been adopting comprehensive 3D printing services as part of their medical practices because they recognize the additional value this approach adds to personalized patient care.^20^, ^21^, ^22^, ^23^, ^24^, ^25^ In particular, for the purpose of personalized patient care, it is critical to design 3D‐printing devices for the construction of patient‐specific anatomical models that are sufficiently accurate and physically safe enough to receive regulatory clearance.^26^ It is also important to develop 3D‐printable nontoxic, biocompatible, lightweight, and flexible soft materials that can provide the required anatomical precision for personalized purpose.^27^, ^28^, ^29^

Unfortunately, most of the conventional and readily available plastics that can be printed using a fused‐deposition‐modeling‐based 3D printer are thermo‐softening plastics such as thermoplastic polyurethane (TPU), polylactic acid (PLA), acrylonitrile butadiene styrene (ABS), and polyvinyl alcohol (PVA). The relatively high elastic moduli of these materials impose a limitation on the fabrication of flexible objects (TPU: 1.31–2.07 GPa, PLA: 65 MPa, ABS: 1.1–2.9 GPa, and PVA: 0.25 GPa^30^, ^31^, ^32^), which, in turn, hinders the fabrication of a structure with high‐resolution anatomical features. One method to make printed plastic materials flexible is to use geometrically optimized lightweight structures such as lattice‐ or network‐type interconnected mesh structures.^33^, ^34^ Another method is to perform 3D printing using softer materials such as liquid supported by granular gel.^35^ In particular, the process of rapid liquid printing (RLP) is advantageous in the preparation of flexible and lightweight materials in a relatively short time (i.e., printing of a complex‐shaped object with dimensions of 6″ × 6″ × 8″ in under 5 min.^36^ However, RLP remains incapable of providing a satisfactorily high resolution (i.e., spatial resolution < 100 µm) because of the diffusion of the printed material into the supporting granular gel. In addition, most of the 3D‐printable soft materials^37^, ^38^, ^39^, ^40^ or mesh‐type structures^41^, ^42^ have poor conductivity, which degrades signal transduction and consequently limits the range of biosensors to which they can be applied.

In this paper, we introduce a pairing of a previously unidentified 3D‐printing approach and soft materials to achieve not only high‐resolution printed features and flexibility of the 3D‐printed materials but also its low density and substantial electrical conductivity. The developed technique and materials were applied to the fabrication of patient‐specific wearable devices such as wearable active and/or passive biosensors intended for use in personalized health monitoring and point‐of‐care diagnostics. The goals of low density and flexibility were achieved via fabrication of 3D microcellular network‐type interconnected conductive materials that could be readily printed using a commercially available inkjet head. High‐resolution 3D scanned data were acquired, using which on‐demand personalized wearable sensors made of the 3D‐printed soft conductive materials were fabricated. These fabricated sensors successfully measured both actively changing bio signals (i.e., a strain sensor for human body movement) and/or passively changing signals, e.g., electromyography (EMG), electrodermal activity (EDA), and electroencephalogram (EEG) signals. Because of the accurately tailored subject‐specific shape of the developed sensors, they showed high‐sensitivity, high‐precision, and real‐time sensing performance in the monitoring of rapidly changing human body signals. The newly developed 3D‐printing technique and materials have wide applicability to various types of wearable, flexible, and lightweight biosensors targeted for use in a variety of inexpensive on‐demand and personalized point‐of‐care diagnostics.

As illustrated in **Figure** **1**a, the high‐resolution 3D printing of soft materials aimed for the realization of wearable sensors began with high‐resolution 3D scanning using coordinated cameras (leftmost upper panel of Figure 1a; also see Figure S1, Supporting Information). Using the detailed information obtained with the 3D scanning system, we could generate anatomically accurate body‐shape data, which could then be used as the input for a 3D printer (leftmost lower panel of Figure 1a). Next, using the inkjet head of the 3D printer and the input personalized body‐shape data, we prepared a tailor‐made porous scaffold via a 3D powder bed printing (3D PBP; see Figure 1a and Figure S2, Supporting Information). The high‐precision printing for conformal contact with specific human skin is a key factor to enhance the sensitivity of the sensor. For the 3D printing, we used a commercially available inkjet head (spatial resolution of 42 µm). Further, we employed typical commercially available typical sugar grains of various grain sizes as the powder in the 3D PBP process (scanning electron microscope (SEM) image of Figure 1b). When printed, the sugar grains formed a 3D cellular‐network‐type interconnected porous structure, which functioned as a scaffold that accommodated conductive nanomaterial. The feature of the porous geometry, such as the porosity (ϕ_0_) and pore size, could be easily controlled by tuning the size of the sugar grains. In the present study, we used three different grain sizes [small (S, 0.01–0.1 mm), middle (M, 0.1–0.3 mm), and large (L, 0.4–0.6 mm)] to control the porosity and pore size (Figure S3, Supporting Information). For instance, as shown in the SEM image in Figure 1b and Figure S4, Supporting Information, ϕ_0_ decreased with increasing grain size (i.e., ϕ_0_ decreased from 0.736 to 0.607). As a template material, sugar grains have several advantages such as cost‐effectiveness compared to conventional polymer filament or powder and environmental friendliness resulting from the high water solubility of the grains. This high water solubility is also advantageous for dissolution of the water‐soluble scaffold via post‐treatment to obtain the final 3D‐printed object. Furthermore, the stickiness and beneficial interconnection of the sugar grains also facilitated formation of a 3D open cellular structure (OCS) into which elastic fillers could be readily loaded. This could be achieved via injection of silicone elastomers such as Ecoflex and poly(dimethylsiloxane) (PDMS) into the porous regions of the scaffold structure and subsequent dissolution of the sugar scaffold (lower SEM image of Figure 1b). Silicone elastomer was chosen because of its high chemical resistance, high compression set resistance, and low elastic modulus. We carefully optimized the Ecoflex:PDMS mixing ratio in order to control the fill‐up dynamics of the elastomer into the scaffold, surface properties, and elastic properties (Figure 5a,b, Supporting Information). As can be seen from the comparison images in Figure 1c, the sugar‐grain scaffold successfully and effectively served as a guiding template for the fabrication of silicone elastomeric porous structures of desired shapes. Finally, to impart conductivity to the porous structure with the eventual aim of realization of a 3D‐OCS‐based conductive and flexible materials, the surface of the porous elastomer structure was coated with single‐walled carbon‐nanotube (SWCNT)‐dispersed acetone solutions. Figure 1d schematically depicts the multiscale hierarchy of the materials used for the realization of the conductive 3D OCS. In particular, to ensure stable dispersion, they were surface‐modified with supramolecular 2‐ureido‐4[1H] pyrimidinone (UPy) groups (UPy‐SWCNTs; see Figure 1d). The acetone solution containing a dispersion of UPy‐modified‐SWCNTs could be coated onto the surface of the optimized 3D porous elastic scaffold in a conformal manner given the fact that the UPy group reduces the interface free energy between the SWCNTs and the silicone elastomer. The conformal coating quality of the SWCNTs‐dispersed acetone solution was confirmed as shown in Figure S6 in the Supporting Information. Because of the conformal coating of the SWCNT‐dispersed acetone solution, the SWCNTs effectively formed a percolation network that imparted electrical conductivity to the porous structure as shown in Figures S4 and S7 in the Supporting Information [typical resistance of the SWCNT‐coated elastic scaffold was **≈**4 kΩ (50 × 10 × 3 mm^3^)]. The 3D OCS elastic structure that is surface‐coated by SWCNTs also showed lightweight property of a density of 0.25 g cm^−3^, that is a quarter of a density of polystyrene foam (i.e., 1.04 g cm^−3^ 43, 44). In terms of the advances of materials, it is important to note that the prepared 3D OCS‐based micro network structures work for flexible, lightweight, conducting, high spatial resolution, water‐resistant, and piezoresistive soft materials. These advantages result from the combination of percolation network of SWCNTs stable supported by the elastic and porous structures.

**Figure 1:**
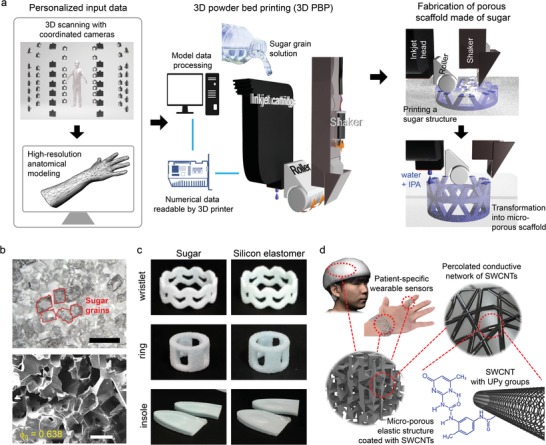
A paring of 3D printing process and printable materials for advanced wearable sensors. a) Schematic illustration of design process of 3D flexible and conductive porous materials using 3D scanning and 3D PBP. b, top) optical microscopy (OM) image of sugar grains and bottom) SEM image of 3D‐printed porous silicone elastomeric structure (black scale bar: 500 µm and white scale bar: 200 µm). c) Comparison between 3D‐printed sugar scaffolds of various shapes and corresponding silicone elastomeric final products. d) Schematic of hierarchical structure of 3D‐printed object and conductive network.

To prepare a conductive yet stimuli‐sensitive surface of a 3D OCS material, it is desirable to employ a minimum amount of conductive filler. In this regard, 1D conductive fillers are advantageous than 2D fillers for the formation of a percolation network. We compared the percolation characteristics of two different types of conductive fillers, SWCNTs (1D) and reduced graphene oxide (rGO, 2D) flakes, as shown in **Figure** **2**a,b. Essentially, because of the dimensionality of the SWCNTs and rGO flakes, both the SWCNT network and the rGO network formed on the surface of the elastic medium were expected to show power‐law dependences of the critical number density as a function of the filler size (i.e., the length for the SWCNTs and the diameter for the rGO flakes) with an exponent of −2.^45^ We confirmed these dependences via 2D percolation simulations (Figure 2a,b). In particular, because of the characteristic geometry of SWCNTs (i.e., stick‐like structure^46^), the critical number density of the SWCNT network for the formation of a percolation network was higher than that of the disk‐like rGO network^47^ (Figure 2c). However, when the critical number density was converted to the areal density, the SWCNT network (which was assumed to be composed of a single SWCNT with a 2 nm diameter) showed much lower, power‐law‐dependent densities as a function of the length scale, which is clearly different from the density results of the rGO network (Figure 2d). Therefore, given the effectiveness of the 2D random network as a conductive percolation network, it was more advantageous to use SWCNT network than rGO network. In addition, the SWCNT network was more beneficial than the rGO network in that the intrinsic tunneling resistance among the individual SWCNTs was higher than that among the individual rGO flakes. Typically, the intrinsic tunneling resistance (*R_i_*) is sufficiently greater than the intrinsic molecular resistivity of a single homogeneous conductive filler, and therefore, it is reasonable to consider *R_i_* as intrinsic resistance of the percolation network of the fillers. This comparison indicates that the lower resistance of the percolation network, the lower its impedance and, consequently, the lower the signal‐to‐noise ratio (SNR). Therefore, it was reasonable to employ the SWCNTs as the conductive filler in the present study.

**Figure 2:**
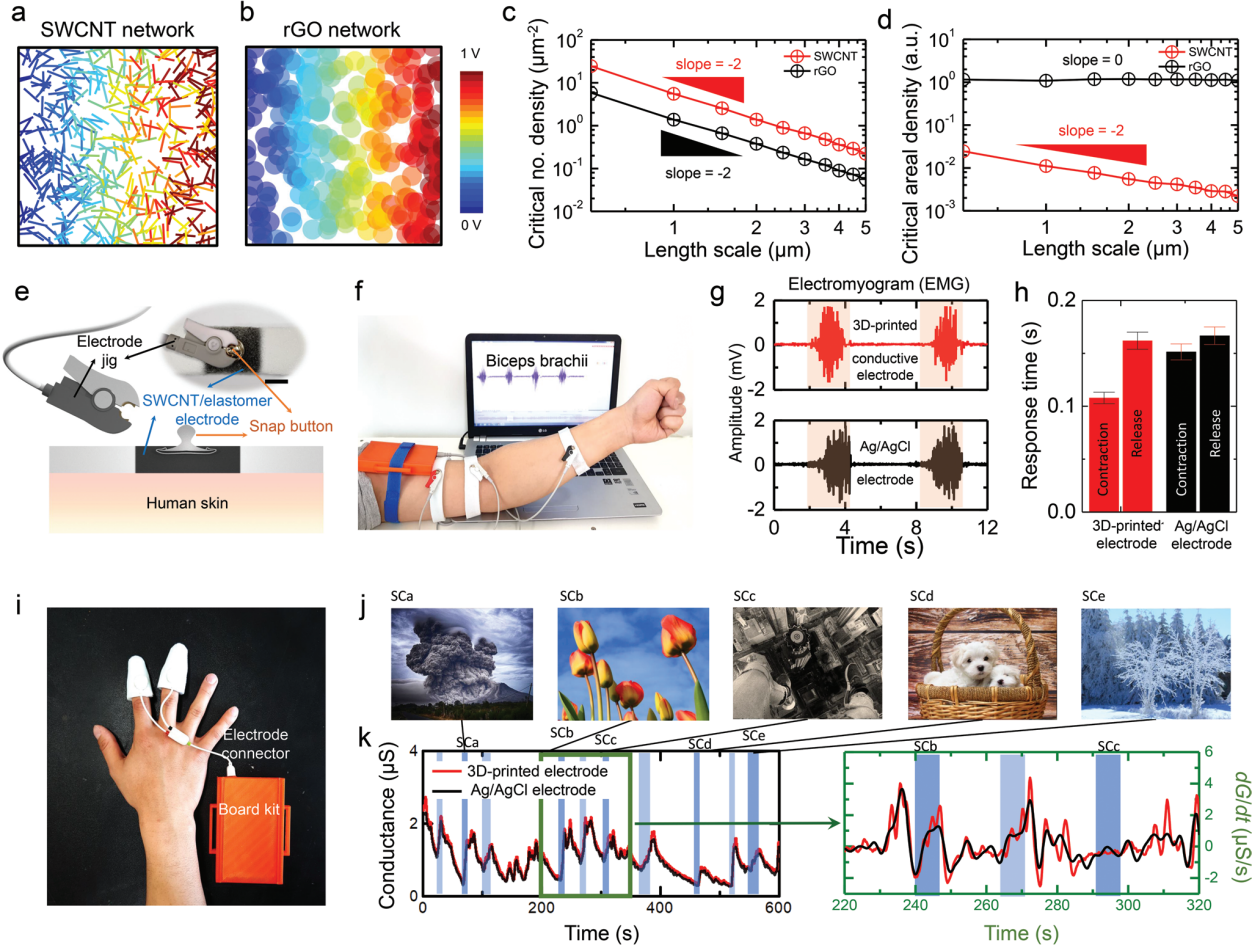
Percolation network of conducting materials for the fabricated passive sensors. a) Simulated networks of SWCNTs (modeled to be stick‐like in shape, with 5 µm length and 2 nm diameter) in 2D square box (box size = 100 µm^2^), having percolated morphology. To visually illustrate the percolated morphology, the spatial distribution of potential is depicted using color (maximum = 1 V on the right‐hand side connected to the cathode, minimum = 0 V on the left‐hand side connected to the anode). b) Simulated networks of rGO flakes (modeled to be disk‐like in shape, with 5 µm length and 2 nm diameter) in 2D square box. c) Plots of critical number densities of SWCNT and rGO networks for formation of percolation network as a function of length scale. d) Plots of areal densities of SWCNT and rGO networks as a function of length scale. e) Schematic setup of biosensing electrode. The inset shows a photographic image of the device (scale bar: 1 cm). f) Photographic image of measurement setup for EMG signals. g) Results of EMG signal measurement using Ag/AgCl electrode and 3D‐printed conductive electrode. h) Comparison of response times of 3D‐printed conductive electrode and Ag/AgCl electrode. i) Photographic image of measurement setup for EDA signals. j) Series of images included in video clip for EDA data acquisition. k, left) results of EDA signal measured using 3D‐printed conductive electrode and Ag/AgCl electrode and right) time‐domain‐differentiated graph of EDA signal measurement results.

The prepared 3D‐OCS‐based flexible and conductive material could be readily tailored as a human‐wearable sensor. As shown in Figure 2e, to convert the 3D OCS to a wearable sensor, a metal snap button was attached to the conductive porous elastomer. Then, as shown in Figure 2f, the metal‐button‐attached flexible patch was connected to a commercially available board kit (Figure S8, Supporting Information) for the measurement and (Bluetooth) transfer of electroencephalogram (EEG), electrodermal activity (EDA), electromyography (EMG) signals. For instance, EMG measures electrical activity of a specific muscle. EMG data were acquired using three flexible electrode‐attached patches affixed to a human forearm in order to facilitate comparison of the different movements of the brachioradialis and biceps brachii muscles (Figure 2f). Signals corresponding to contraction and relaxation of the muscles were measured in real time through the affixed patches. When skeletal muscle is contracted, motoneurons generate electrical impulses and action potential appears. As subjects contract their muscles harder, more motoneurons are excited, resulting in high action potential and voltage signal. As shown in Figure 2g, the maximum contraction of the muscles (i.e., amplitude of 0.8 mV generated between 8 and 10 s) was successfully detected, which was four times the amplitude of the measured signals of the muscles at rest. As compared in Figure 2g and Figure S9 in the Supporting Information, it is noteworthy that the 3D‐printed conductive patch exhibited substantially enhanced sensing performances of the muscle contraction to those measured by a reference wearable sensor with conventionally available Ag/AgCl electrodes. Although the EMG sensors coupled with both electrodes successfully detected the state changes in the muscle movements, the 3D‐printed conductive patch‐based sensors showed notably higher sensitivity than the Ag/AgCl electrodes (Figure S9, Supporting Information). Using a wavelet denoising method and a decomposition algorithm with a maximum variance change point algorithm, we calculated SNR for the EMG signals, and found that SNR of 12.87 dB for the 3D‐printed conductive patch‐based sensors, that was significantly higher than SNR of 9.68 dB for the reference sensor with the Ag/AgCl electrodes (see detailed algorithm in Supporting Information (SI). Furthermore, the 3D‐printed conductive‐patch‐based sensor responded faster to a particular muscle movement than did the reference sensor (Figure 2h).

In addition, we proceeded to compare EDA data acquisition performances of the two sensors. It is well known that emotional stimulation or unconscious mental processing leads to a change in the electrical conductivity of human skin, which is caused by automatic‐nerve‐induced variation of the electrical properties of the skin. This change in electrical conductivity can be recorded and tracked in real time by measurement of EDA. For this measurement, as shown in Figure 2i, we fabricated 3D‐printed thimble‐shaped conductive‐patch‐based sensors, which fit over individual‐specific index and middle fingers with high precision (i.e., fitting error <50 µm). Emotional stimulation was induced by making a human subject watch a 10‐min video clip that was designed to trigger a variety of emotional stimulations (i.e., Figure 2j; also refer to Figure S10, Supporting Information, for the complete clip sequence). As shown in the left‐hand plot of Figure 2k, the emotional transition of the subject was detected and tracked in real time by the 3D‐printed patch‐based EDA and the reference EDA sensors. As can be seen from the EDA signal profiles, the two sensors showed similar conductance burst patterns, which were in accord with the designed stimulations sequence. Notably, the 3D‐printed conductive patch‐based EDA sensors showed higher sensitivity than the reference EDA sensor with Ag/AgCl electrodes (Figure S11, Supporting Information). Using a wavelet denoising method, we calculated SNR for the EDA signals, and found that SNR of 31.38 dB for the 3D‐printed conductive patch‐based sensors, whereas the reference sensor showed SNR of 27.47 dB. To systematically compare the sensing performances of these two EDA sensors, we further analyzed the temporal difference of the EDA signals. As shown in the right‐hand panel of Figure 2k (and the complete set of data in Figure S12, Supporting Information), the 3D‐printed patch‐based EDA sensor appeared to be more sensitive to the transition of emotional stimuli than the reference sensor. For instance, the 3D‐printed patch‐based EDA sensor showed 1.12–1.45‐fold higher SNR for the stimuli provided at 228.2 and 262.2 s. The stimuli provided at 303.7 s was detected only by the 3D‐printed patch‐based EDA sensor. These observations indicate that the 3D‐printed patch‐based sensor can serve as a more sensitive and reliable wearable patient‐specific EDA sensor.

We further examined the sensing performance of the 3D‐printed patch‐based sensors during the detection and tracking of brain EEG signal in real time. EEG records electrical activity of the brain. By measuring potential fluctuation resulting from ionic current within the brain neurons, EEG gives a clue for neurological conditions. As shown in **Figure** **3**a, a head‐mountable and over‐ear‐wearable 3D‐printed patch‐based EEG sensor was prepared using three electrodes: two of these electrodes were attached to the forehead (ground and positive electrodes) and the third electrode was attached under the earlobe. The wearable sensor measured real‐time EEG signals for three different sleep phases: non‐rapid eye movement (non‐REM) sleep phase, REM sleep phase, and comfortable (comfy) phase. As is evident from the original EEG data in Figure 3b, the 3D‐printed patch‐based EEG sensor was able to successfully distinguish the different sleep phases. For a detailed quantitative comparison, the acquired original EEG data were systematically analyzed in the frequency domain by 1D fast Fourier transform (FFT) and multilevel 1D wavelet decomposition. As depicted in the uppermost row in Figure 3c, the 3D‐printed patch‐based EEG sensor effectively differentiated the three different sleep phases in the frequency domain. In particular, transformed EEG data demonstrated the different intensities of the five brainwaves (i.e., delta (δ) waves in the 0–4 Hz range, theta (θ) waves in the 4–8 Hz range, alpha (α) waves in the 8–12 Hz range, beta (β) waves in the 12–30 Hz, and gamma (γ) waves in at 30 Hz or higher frequency^48^) in the three different sleep phases. A more detailed comparison could be performed via wavelet decomposition of the data. From the wavelet‐decomposed EEG data in the second to sixth rows in Figure 3c, we found that the 3D‐printed patch‐based EEG sensor clearly detected the five brainwaves in the three different sleep phases. For instance, the δ waves peaked in the non‐REM sleep phase, whereas the θ waves peaked in the REM sleep phase. In contrast, in the comfy phase, the intensities of the γ and β waves were notably higher than those of the other waves. When the subject was in the sleep phase, the wavelet peak gradually shifted to a high frequency. From these experimental examinations of the 3D‐printed patch‐based biosensors used to detect various types of signals (EMG, EDA, and EEG), we can conclude that the fabricated sensors serve as reliable, general‐purpose, individual‐specific wearable, and highly sensitive passive biosensors.

**Figure 3:**
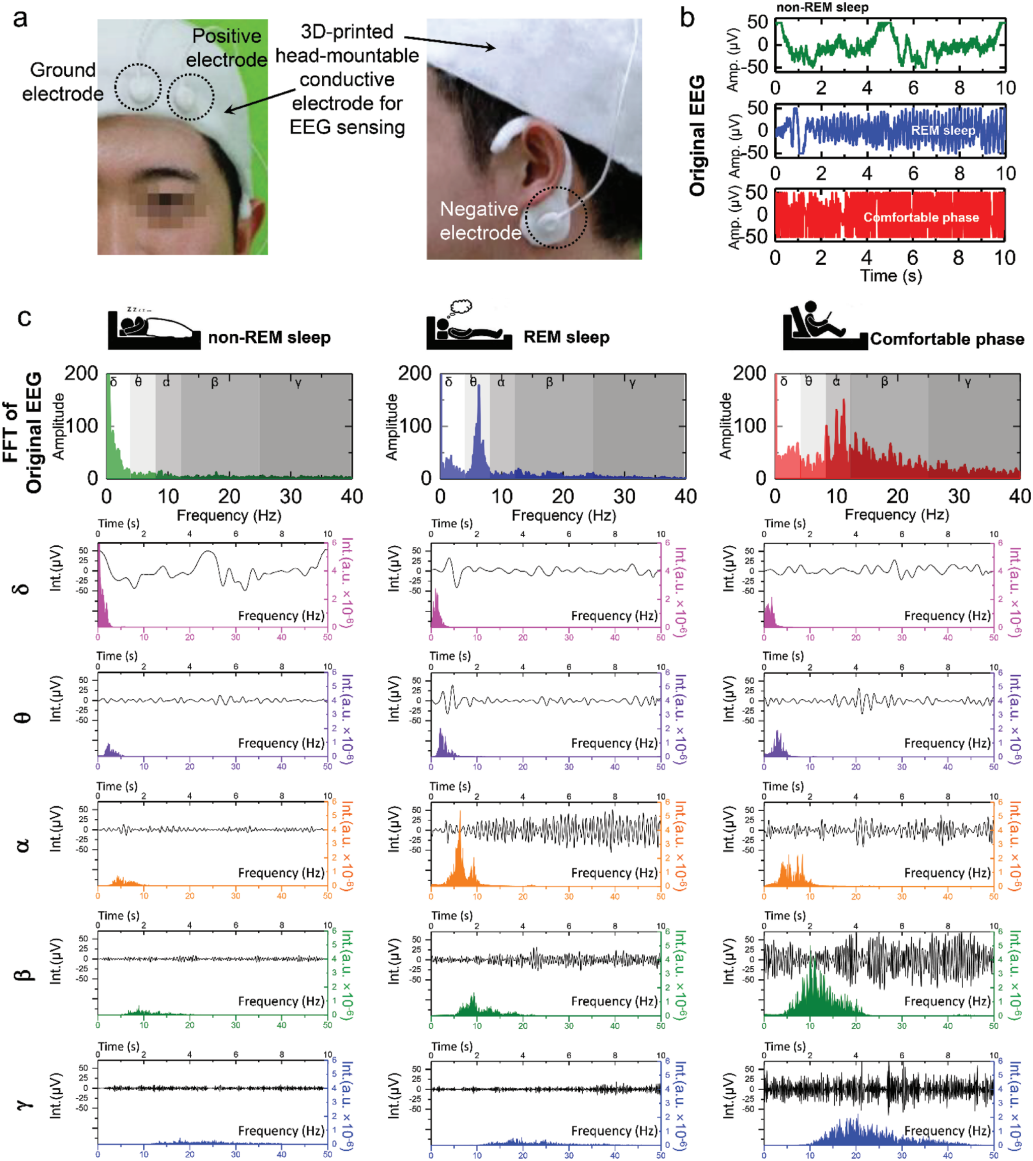
High‐precision and personalized EEG signals measured by wearable sensors. a) Photographic image of measurement setup for EEG signals. b) Measured EEG data of subject in each sleep phase. c) Processing results of EEG signals in each phase. The left, middle, and right‐hand columns show the FFT results of raw signals, wavelet signals in different brainwave regions, and FFT results of wavelet signals, respectively.

In addition to the above‐described passive‐type wearable biosensors, piezoresistive on‐body‐strain sensors, which can be applied to active‐type wearable biosensors, could also be fabricated by taking full advantage of the properties of the 3D conductive and flexible porous material. To realize sensitive strain sensors, it is critical to employ flexible materials that show a sufficiently high gradient of the relative change in resistance with the applied strain. Here, we found that the SWCNT‐coated silicone elastomeric 3D OCS effectively functioned as a piezoresistive strain sensor with a sufficient gauge factor (GF). We also found the SWCNT network to be more favorable than the rGO network for achieving a high GF (refer to SI. 1 and Figure S13a, Supporting Information). This advantage of the SWCNTs over rGO flakes is partially attributed to the fact the SWCNT network is more beneficial than the rGO network in that the intrinsic tunneling resistance among the individual SWCNTs is higher than that among the individual rGO flakes. Given the fact that the tunneling distance and the pathway density depend on the external strain, the tunneling resistance varies under application of external stress. Therefore, it is preferable to use multiple fillers with a higher tunneling resistance, which provide higher strain sensitivity. Higher tunneling resistance can be achieved using fillers with a higher potential barrier, which is equivalent to the work function of the neighboring fillers separated by the surrounding medium. The work function of SWCNTs ranges between 4.95–5.95 eV,^49^ whereas that of the rGO flakes is lower (i.e., 0.2–0.7 eV^50^). Therefore, it was reasonable to employ percolation network of SWCNTs, instead of the rGO network, for achieving higher strain sensitivity.

To further understand the materials properties of the SWCNT‐network‐coated elastomeric 3D porous network structure for the realization of strain sensors, we constructed a simple mathematical model to determine the relationship between the GF and the strain. By examination of simulated porous networks (Figure S13b, Supporting Information), the effective resistivity of the entire porous structure (i.e., the 3D OCS with the interconnected pillars coated with the SWCNT network), ρ_*T*_, relative to the effective resistivity of the SWCNT‐network‐coated pillars in the porous materials, ρ_*S*_, can be expressed as a function of the porosity of the 3D OCS, *f*, i.e., $\frac{\rho_{T}}{\rho_{S}}={\left(\frac{1\;+\;\phi }{1-\;\phi }\right)}^{2}$. With this approximation, we can derive a simple relationship between the relative resistance ($-\Delta R_{T}/R_{0}$) of the percolation network of SWCNTs on the surface of the 3D elastic OCS and the compressive strain (ε) as follows (for the detailed derivation, refer to SI. 1):

(1) $$\begin{array}{l} \frac{\Delta R_{T}\left(\varepsilon \right)}{R_{T0}}\;\approx \;\left(1\;-\;\nu \left(\varepsilon \right)\varepsilon \right){\left(\frac{1\;-\;\phi_{0}}{1\;+\;\phi_{0}}\right)}^{2}\exp\left(-\gamma d_{0}\nu \left(\varepsilon \right)\varepsilon \right){\left[\frac{1\;+\;\phi_{0}\exp\left[-f\left(\nu \right)\varepsilon \right]}{1\;-\;\phi_{0}\exp\left[-f\left(\nu \right)\varepsilon \right]}\right]}^{2},\\ \;f\left(\nu \right)\;=\;\frac{1\;+\;\nu \left(\varepsilon \right)}{3\left[1\;-\;\nu \left(\varepsilon \right)\right]},\;\nu \left(\varepsilon \right)\;=\;\nu_{0}\;+\;\alpha \varepsilon \end{array}$$

where φ_0_ is the initial porosity of the 3D OCS, ν_0_ is the characteristic Poisson's ratio of the elastic medium, *γd*_0_ is the tunneling resistance parameter, and α is a constant. From Equation (1), it is evident that the relative resistance increases with increasing porosity of the 3D OCS (Figure S14a, Supporting Information). The strain sensitivity of the 3D‐OCS‐based elastic and conductive material can be further maximized by optimizing the surface and elastic properties of the 3D OCS. As can be seen from the analysis in SI. 2 and the data in Figure S14b, Supporting Information, it was advantageous to use an Ecoflex‐rich elastic medium with an optimized composition, which would ensure a more uniform surface distribution of the SWCNTs and subsequently result in an isotropic, flexible, and uniform strain‐responsive resistance of the sensor material. Furthermore, the operational stability of the sensor was investigated during 3000 bending cycles under 25% strain as shown in Figure S15, Supporting Information. The sensor exhibited the excellent reliability and durability.

Using the optimized 3D‐OCS‐based flexible and conductive material, we fabricated strain sensors for individual‐specific strain measurement, which were targeted at a point‐of‐care purpose motion controller. First, as shown in **Figure** **4**a, when a ring‐shaped 3D‐printed strain sensor was used, the bending strain of the subject's index finger could be accurately and systematically determined from the change in the current level, since these changes were linearly proportional to the bending angle. Second, as shown in Figure 4b, the change in the current level with the bending angle of the subject's wrist was successfully measure using a wristlet‐shaped 3D‐printed strain sensor. In a similar manner, the bending strain of the subject's knee was successfully measured using a knee‐band‐shaped 3D‐printed strain sensor affixed around the knee, as depicted in Figure 4c. Without any reference to the corresponding body parts, the 3D‐printed strain sensors accurately detected a single universal relationship between the bending strain and the change in the current level (i.e., a slope of 1.7 × 10^−2^ deg^−1^; see Figure 4d). This indicates that the fabricated 3D‐printed strain sensors functioned in a reliable and accurate manner and could therefore be used for general purpose. In addition, the fabricated strain sensors exhibited satisfactory real‐time monitoring performance with fast response times [i.e., characteristic time scales for strain initiation (τ_on_ = 6.1 × 10^−3^ s) and strain termination (τ_off_ = 8.5 × 10^−3^ s); see Figure 4e]. The characteristic times scales were calculated by fitting the current change curve with a single exponential function. Apart from being used for simple monitoring of bending strain of the human body, the 3D‐printed strain sensor could also be employed for monitoring of signals of translational motion of the human body. For instance, when integrated with a shoe insole, the 3D‐printed strain sensor was found to be capable of tracking translational motions in real time, as shown in Figure 4f. In particular, the sensor promptly tracked the translational motions and successfully distinguished three different types of motion (i.e., walking, race walking, and running) without any accumulation of current change. Because of the high‐resolution 3D printability of the 3D‐OCS‐based conductive and flexible material of the strain sensor, the sensor could be readily integrated with an individual‐specific shoe insole (Figure S15, Supporting Information). In a similar manner, the 3D‐printed strain sensor could also be applied as a pressure sensor (Figure S16, Supporting Information).

**Figure 4:**
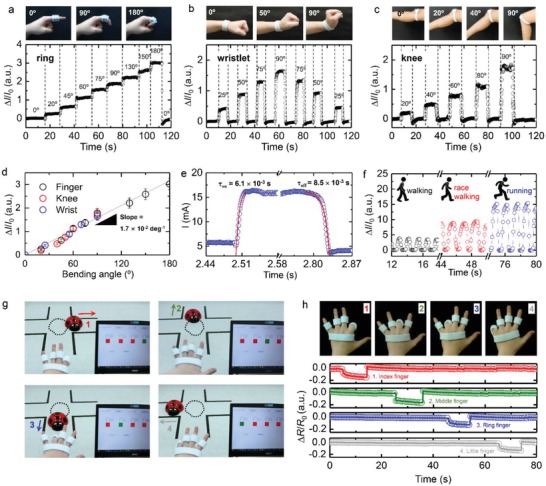
High‐precision, high‐resolution, and personalized wearable active sensors and motion controller. Real time measurement with the normalized current results for a) ring‐shaped, b) wristlet‐shaped, and c) knee‐band‐shaped strain sensors with specific bending angles. d) Plot of normalized current versus bending angle for different strain sensors. e) Measurement results of response times of strain sensor. f) Results of real‐time measurement of three different translational motions using insole‐type strain sensor. g) Screenshots of Supporitng Video depicting motion of RC car controlled using 3D‐printed wearable motion controller. h, top) photographs showing specific finger‐bending motions assigned to different directions and bottom) graphs showing results of real‐time measurement of normalized resistances during various finger‐bending motions.

Finally, it was possible to fabricate a wearable motion controller by use of the strain sensors. For instance, as shown in Figure 4g and Supporting Video, the motion direction of an object (i.e., N, S, E, and W) could be remotely controlled by bending one of four fingers (excluding the thumb) individually. For this purpose, 3D‐printed thimble‐shaped patch‐based strain sensors with a wireless signal transmitter were fastened around four fingers (excluding the thumb). As shown in Figure 4h, the individual bending of each of the four fingers was accurately tracked and converted into direction changing signals in real time owing to the fast response time (i.e., 5.8 × 10^−2^ s). On the basis of all these demonstrations of strain measurements and control performances, we propose that the novel pairing of the developed 3D‐printing technique and 3D‐printable conductive and flexible material with an SWCNT network can be applied to the fabrication of personalized strain sensors for body‐strain measurement and a remotely operable motion controller.

In summary, we developed a set of a new 3D‐printing technique and 3D‐printable soft materials to achieve high‐resolution‐printed features with flexibility, low‐density, and electrical conductivity of the 3D‐printed materials targeted for patient‐specific wearable devices. The lightweight and flexibility were achieved via fabrication of a scaffold composed of 3D‐printed sugar grains. The scaffold then turned into 3D microcellular network‐type interconnected conductive and elastic materials using SWCNT‐network‐coated silicone elastomer. With high‐resolution 3D body scanned data, personalized wearable biosensors made of the 3D‐printed flexible and conductive materials were fabricated. The fabricated sensors successfully and accurately measured both actively changing bio signals (body strain) and passively changing bio signals (EMG, EDA, and EEG). Because of the accurately tailored subject‐specific shape of the developed sensors, they exhibited higher sensitivity and faster real‐time tracking performances in the monitoring of rapidly changing human body signals. Given the functional multiple advantages, the newly developed 3D‐printing technique and materials are expected to be applied to wide range of wearable, flexible, and lightweight biosensors targeted for use in a variety of inexpensive on‐demand and personalized point‐of‐care diagnostics.

## Conflict of Interest

The authors declare no conflict of interest.

## Supporting information

Supporting Information

Supplemental Video 1
