# Supplementary material for: 3D‐Printed Sugar Scaffold for High‐Precision and Highly Sensitive Active and Passive Wearable Sensors
Source: Adv Sci (Weinh). 2019 Nov 11;7(1):1902521. doi: 10.1002/advs.201902521 (PMC6947489; doi:10.1002/advs.201902521)
Supplement: Supplementary file 1 — Supporting Information [file ADVS-7-1902521-s001.pdf]

Copyright WILEY-VCH Verlag GmbH & Co. KGaA, 69469 Weinheim, Germany, 2019.

Supporting Information

for *Adv. Sci.,* DOI: 10.1002/advs.201902521

3D-Printed Sugar Scaffold for High-Precision and Highly Sensitive Active and Passive Wearable Sensors

Dong Hae Ho, Panuk Hong, Joong Tark Han, Sang-Youn Kim, S. Joon Kwon, and Jeong Ho Cho**

Supplementary Information for

3D-Printed Sugar Scaffold for High-Precision and Highly-Sensitive Active and Passive Wearable Sensors

Dong Hae Ho,[1,†] Panuk Hong,[1,†] Joong Tark Han,[2] Sang-Youn Kim[3], S. Joon Kwon,[4,*] Jeong Ho Cho[5,*]

[1]SKKU Advanced Institute of Nanotechnology (SAINT), Sungkyunkwan University (SKKU), Suwon 16419, Republic of Korea.
[2]Nano Hybrid Technology Research Center, Korea Electrotechnology Research Institute (KERI), Changwon 642-120, Republic of Korea.
[3]Interaction Laboratory, Advanced Research Technology Center, Computer Science and Engineering, Korea University of Technology and Education, Cheonan, ChungNam 330-708, Korea.
[4]Nanophotonics Research Center, Korea Institute of Science and Technology (KIST), Seoul 02792, Republic of Korea.
[5]Department of Chemical and Biomolecular Engineering, Yonsei University, Seoul 03722, Republic of Korea.

*Corresponding authors: cheme@kist.re.kr and jhcho94@yonsei.ac.kr
[†]These authors contributed equally to this work.

Experimental Section

3D body scanning. To fabricate on-demand biosensing devices that fit the subject with high-precision, detailed geometric data of the subject's body were required. Precise geometric data of various body parts were measured via 3D body scanning. As shown in the right-side image in Supplementary Fig. 1a, 3D geometric data of the subject's body were measured using 56 digital single-lens reflex (DSLR) cameras. This 3D scanning technique, known as 3D photogrammetry, is a method that estimates the 3D coordinates of surface points by using photographs of a single physical object taken from different angles. In this study, 56 DSLR cameras photographed the subject standing on a cylindrical platform from all directions, after which all of the captured photographs were entered into software. The software searched for similar surface points in photographs compared side by side and estimated the locations of the cameras. On the basis of information of the camera location and similar surface points detected in every photograph, the exact location of each 3D dot on the subject was calculated. The calculated 3D dots formed the surface of the interpolated mesh of the 3D model. The entire process of acquisition of data for the 3D model took less than 3 h; capture of photographs took about 2 min; finally, combining of all the data to construct the 3D model took 2–3 h. Supplementary Fig. 1c shows the armbands that were fabricated on the basis of the 3D geometric data of the subject's body and that fit the left arm of the subject perfectly. To demonstrate the accuracy of the photogrammetry-based 3D model, the difference between the body geometry estimated using the 3D model and the actual body geometry of the subject was estimated. As shown in Supplementary Fig. 1b, various body parts of the subject were selected and measured. The inset graph in Supplementary Fig. 1b shows the measured length of each body part and the difference between the scanned and measured data. The comparison results revealed that the difference between the data estimated using the 3D model and the measured data was less than 5%. Then, on the basis of the 3D body geometry data acquired by the above-described process, various sugar-based porous structures were fabricated by 3D printing.

3D sugar printing. To fabricate sugar structures, it was crucial to prepare suitable sugar-based ink that possessed appropriate chemical properties. To this end, isopropyl alcohol (IPA)-water solution (mixing ratio of 3:7) was used as the sugar-binding material. IPA serves as a controlling agent for solvent evaporation dynamics and consequently enables rapid printing and achievement of a high resolution. We maintained the printing bed temperature at 70°C in order to control the binder evaporation time to under a few minutes. To achieve the high resolution of the PBP product, it was also important to maintain surface flatness. For this purpose, we used a polytetrafluoroethylene (PTFE) roller, since PTFE is well known as a nonstick surface coating material. The sugar supply system consisted of a sugar reservoir, a transport path, and a shaker motor. The reservoir was positioned on top of the inkjet head and connected to the transport path. The shaker motor was positioned along the vertical transport path (Supplementary Fig. 2). When the shaker motor was triggered on, it caused vibration of the transport path and sprinkling of sugar from the lower end of the vertical transport path onto the printing area. As a result, the sugar in the reservoir moved toward the transport path to occupy the vacant space. All these components were mounted onto a customized printed circuit board. When the inkjet head passed through the designated position, the shaker motor was triggered on and it sprinkled sugar onto the printing area; subsequently, the inkjet head followed the transport path while the roller was activated. The roller flattened the surface and the inkjet head printed the desired pattern on the flattened surface. Finally, the

transport path was shifted slightly to the next position. Fig. 1a shows a simplified schematic of one printing cycle. Several repetitions of this printing cycle (Supplementary Fig. 2) resulted in stacking up of the printed layers to form a 3D object without any supporting structure. The printed object was initially submerged within the surplus amount of sugar in the powder bed, from which it was subsequently removed (Supplementary Fig. 2). There are two key factors that affect the spatial resolution; sugar grain size and droplet size ejected from inkjet head. If sugar size is larger than droplet size of the ink, minimum spatial resolution determined by the size of the sugar size and vice versa. By controlling these variables, we achieved 100 μm of spatial resolution. To fabricate porous elastomer composite, sugar template was submerged into the uncured silicone elastomer and vacuumed. After curing followed by dissolution of the sugar scaffold (Supplementary Fig. 4), we obtained elastomeric scaffold, which has the precisely equivalent shape of the sugar scaffold as shown in Fig. 1d. To make 3D conductive OCS network, SWCNTs or rGO solution were injected into the elastomeric scaffold using a syringe. For the conductive pillar, 1 wt% SWCNTs solution was used. 10g of SWCNTs solution in acetone (Nano Solution, Inc., 10 wt%) was diluted to 90 g of acetone to make 1 wt% SWCNTs solution. The rGO solution was prepared from natural graphite (Alfa Aesar., purity 99.999% - 200 mesh) using the modified Hummer's method.

Electrical measurement. The BITalino (r)evolution board kit was used to measure the EEG, EMG, and EDA signals. EEG used a 3-lead system that electrodes were attached at the frontal pole 1, frontal 7, and left ear lobe. EMG used the same 3-lead system: positive and negative electrodes were attached to the beginning and middle of the biceps brachii, respectively, while the reference electrode was positioned at the brachioradialis. EDA used 2-lead system, where electrodes were attached at the finger phalanges. For comparison, commercial 3M^{TM} Red Dot^{TM} electrodes were used. The active sensing properties were evaluated using Keithley 4200 source-measure units and pulse-measure units with custom-built bending machine.

Experiments on Human Subjects. Electrodiagnosis on human subjects were performed according to IACUC guidelines. All the human subjects were volunteer.

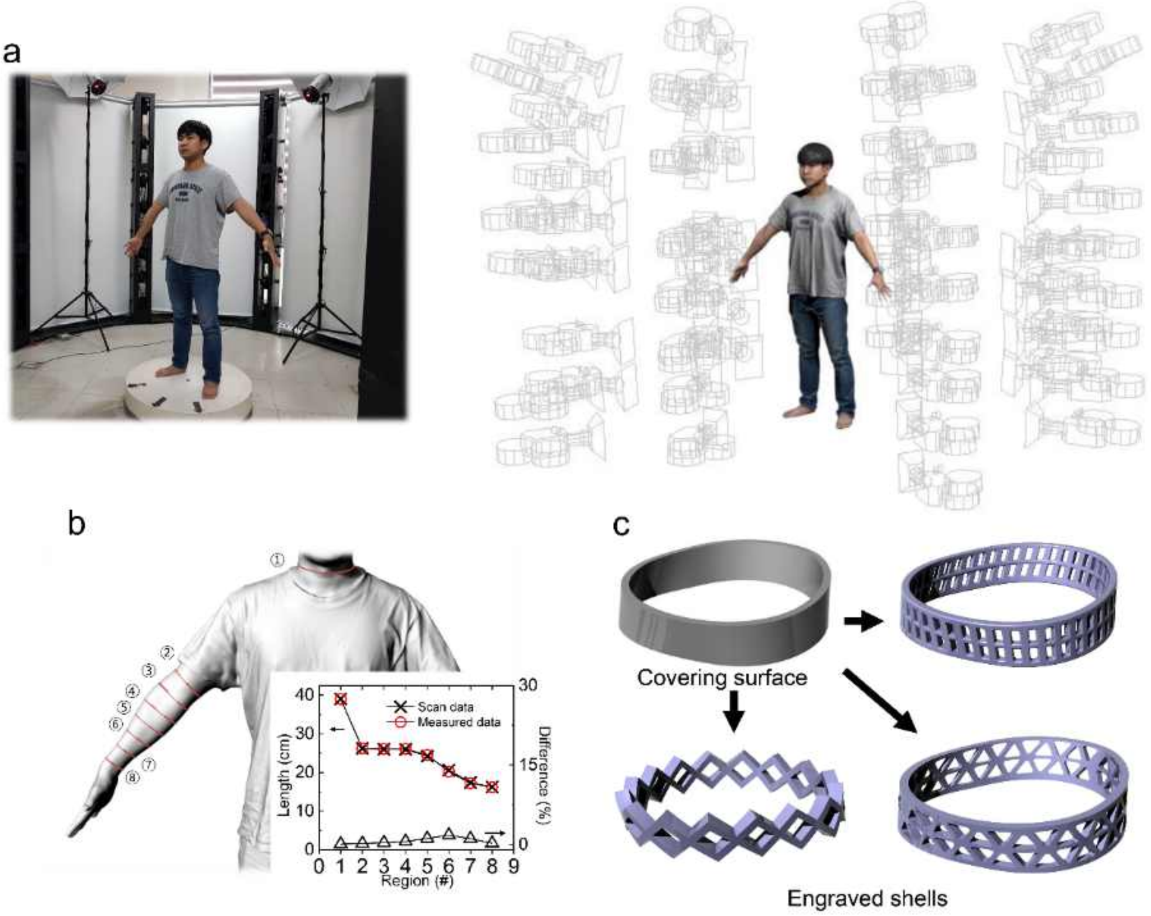

Supplementary Figure 1. **a** Three-dimensional body scanning scheme using coordinated cameras around human subject: (left) actual photographic image of scheme and (right) schematic of illustration of detailed coordinated cameras around human subject. **b** Comparison between the 3D scan data and measured data **c** Various arm band design based on the scanned body geometry data.

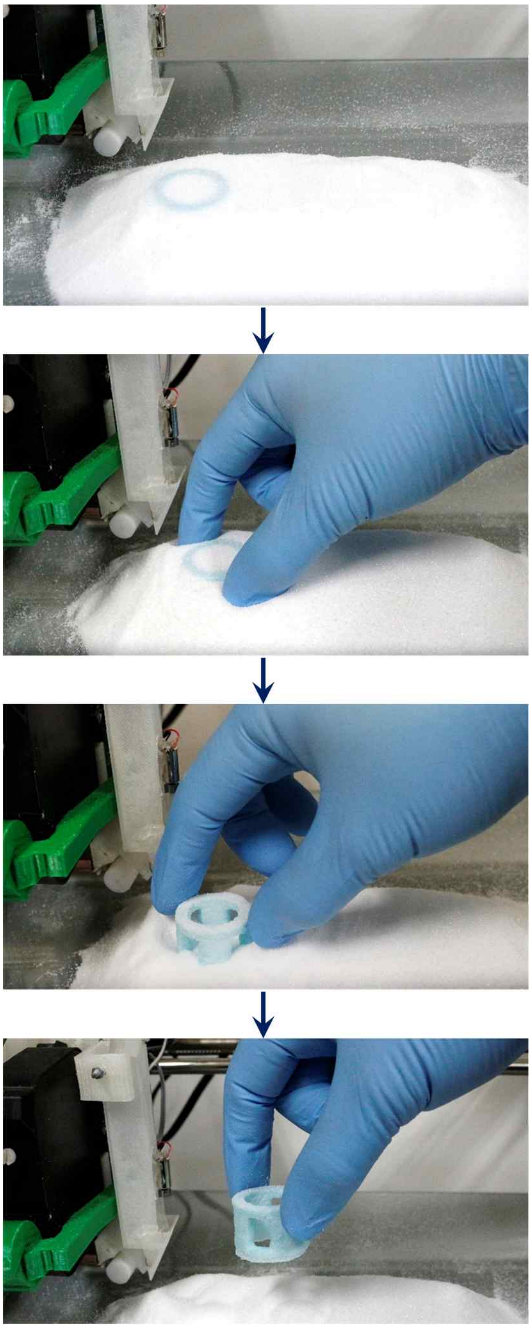

Supplementary Figure 2. A procedure of 3D printing for the fabrication of a structure with 3D-shaped feature composed of sugar grains.

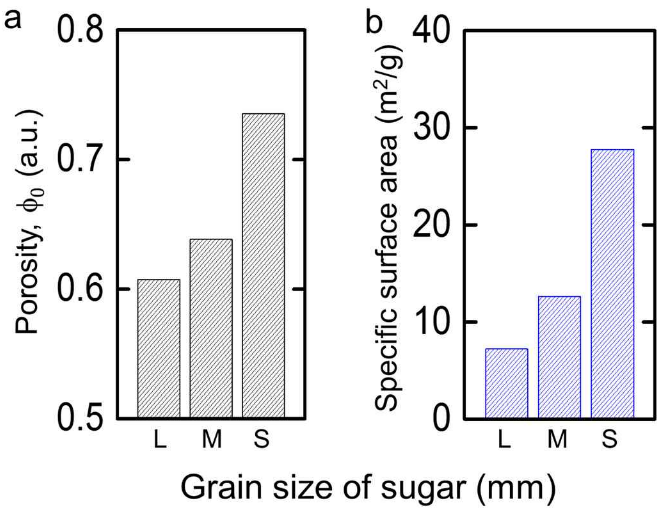

Supplementary Figure 3. a Effects of the sugar grain size on the porosity and **(b)** the specific surface areas of the 3D open cellular structures. For the grain size, S, M, and L denotes small (0.01-0.1 mm), middle (0.1-0.3 mm), and large (0.4-0.6 mm) sizes, respectively.

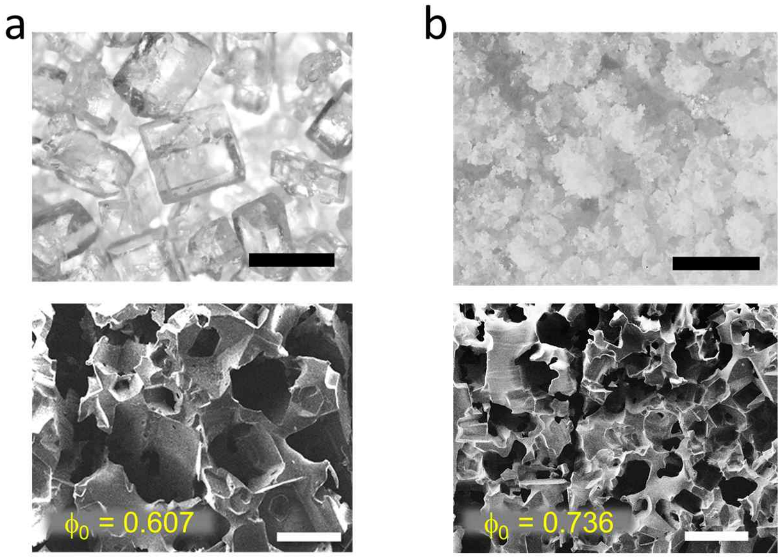

Supplementary Figure 4. Optical microscope (OM) images of 3D-printed sugar-grains (upper panels) and SEM images of the sugar-melted 3D porous structures (lower panels) with different sugar grain sizes (**(a)**: large and **(b)**: small grain sizes).

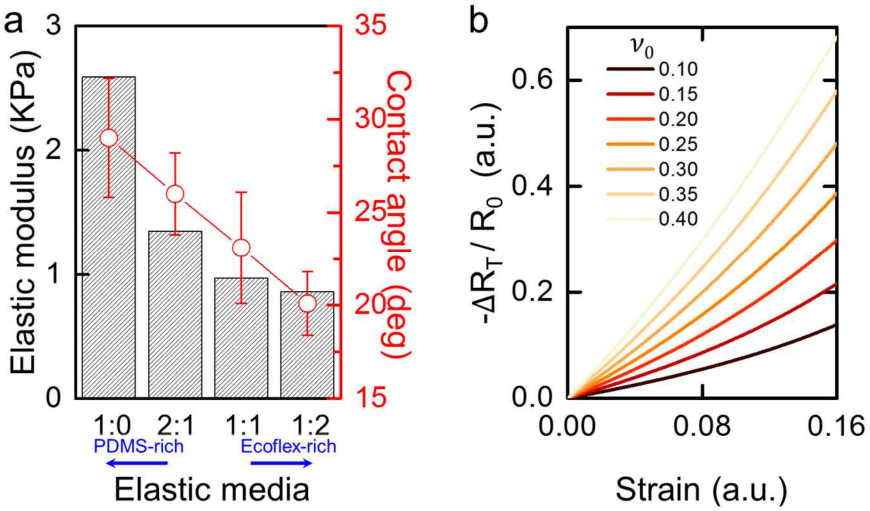

Supplementary Figure 5. a Effects of composition of Ecoflex® on the elastic modulus and the contact angle of SWCNT-dispersed acetone droplet. **b** A relationship between $-\Delta R_T/R_0$ and ε for different values of ν_0.

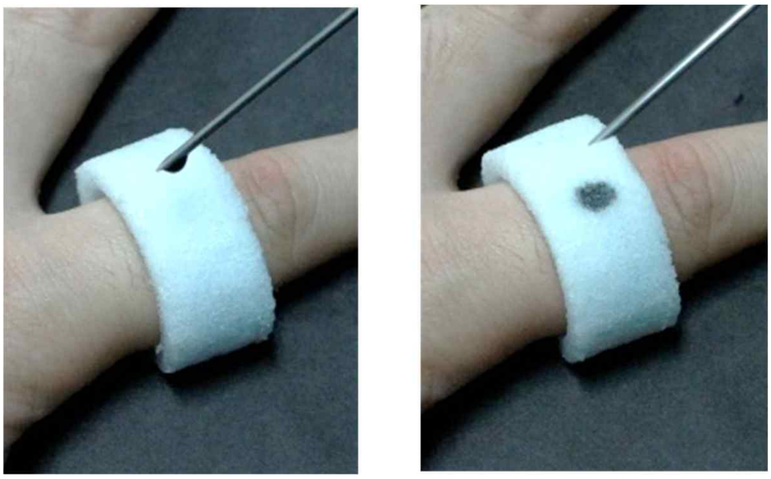

Supplementary Figure 6. Photographs of 3D microporous elastic structures before (left) and after (right) loading SWCNTs-dispersed acetone solution.

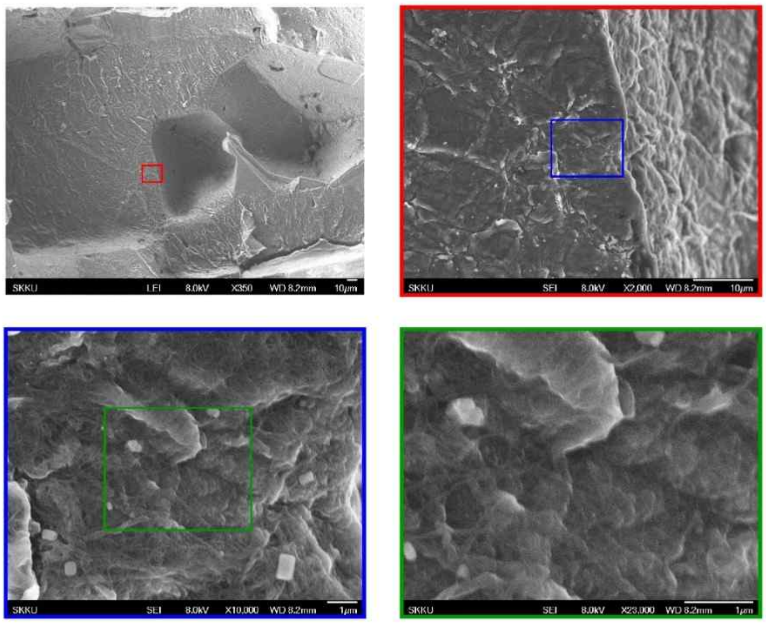

Supplementary Figure 7. Series of SEM images that progressively magnify the surface of SWCNTs conformally coated on porous elastomer structure.

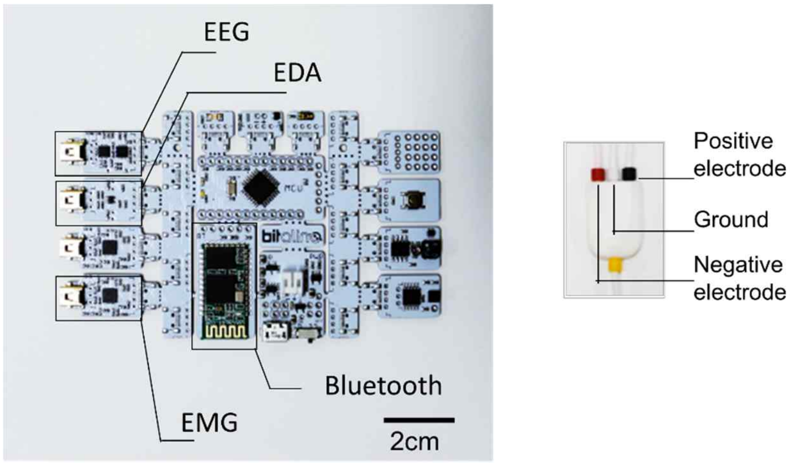

Supplementary Figure 8. (Left) a commercially available board kit which can measure EEG, EMG, and EDA signals, that can be (Bluetooth) connected to the 3D-printed electrodes (right).

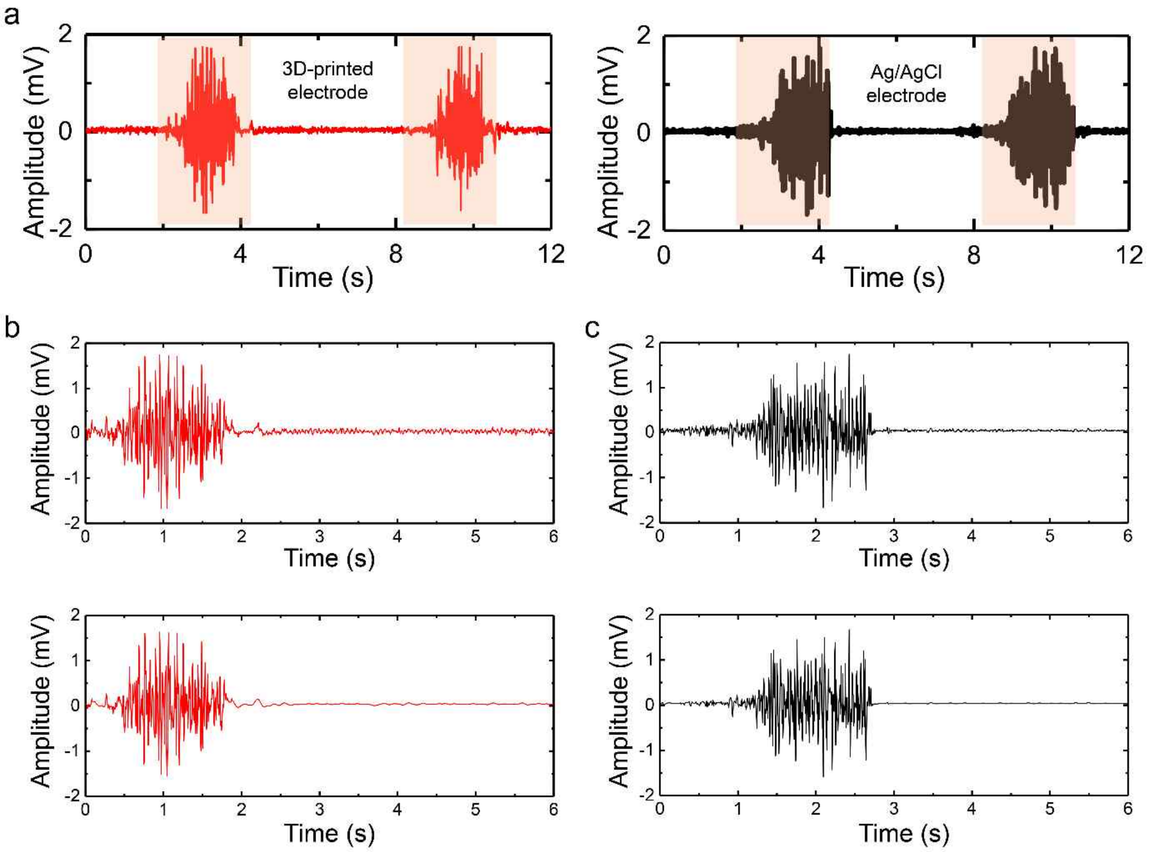

Supplementary Figure 9. a Comparison of EMG signals detected by sensors with the 3D printed electrode (left) and the commercially available Ag/AgCl electrode (right). Raw and wavelet denoised EMG signals measured by the sensors (excerpted from **(a)**) with the 3D printed electrode **(b)** and the Ag/AgCl electrode **(c)**.

Photos	Time
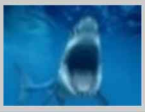	23.8~32s
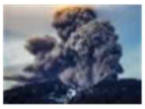	65.3~73.5s
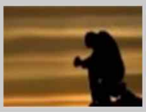	98.1s~113.4s
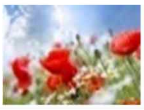	228.2~238.6s
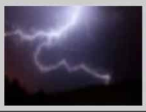	264.3~273s
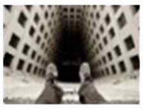	303.7~311.9s
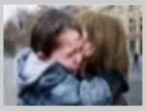	362.7~382.4s
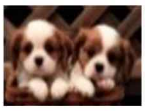	454.7~465.5s
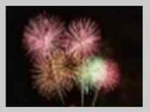	515.8~524s
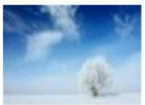	546.3~565.5s

Supplementary Figure 10. A complete sequence of the video clips used for the visual stimulation in the measurement of EDA data.

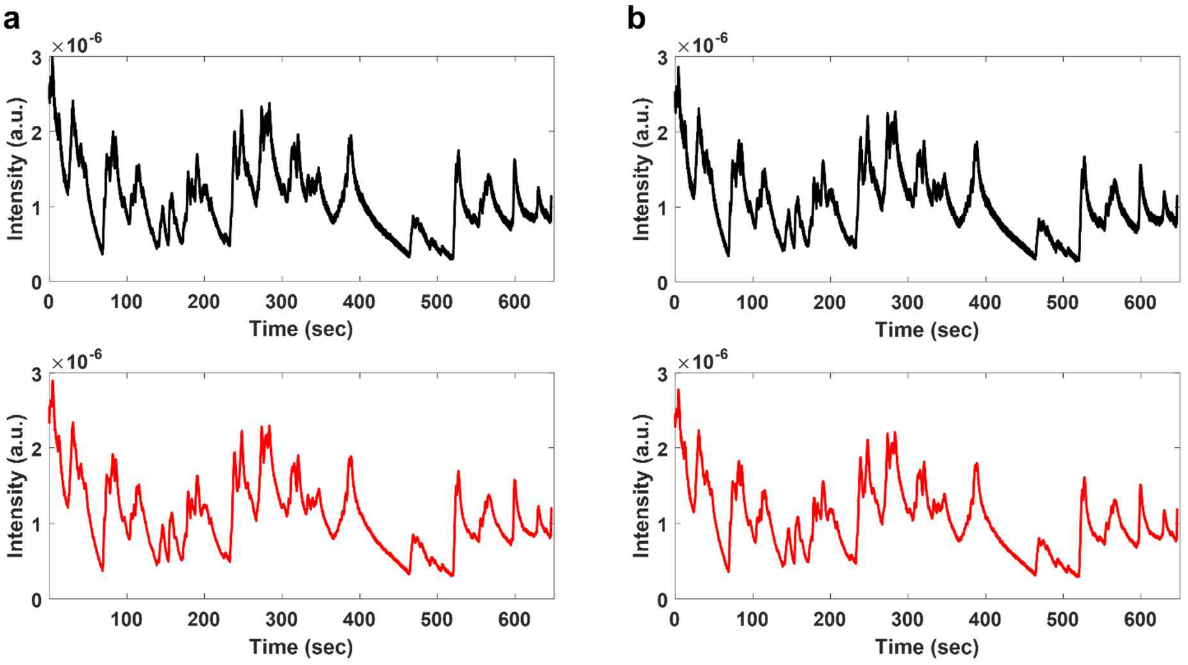

Supplementary Figure 11. Raw (upper) and wavelet denoised (lower) EDA signals measured by **(a)** the 3D-printed conductive patch-based sensor with the 3D printed electrode and **(b)** the reference sensor with commercially available Ag/AgCl electrode.

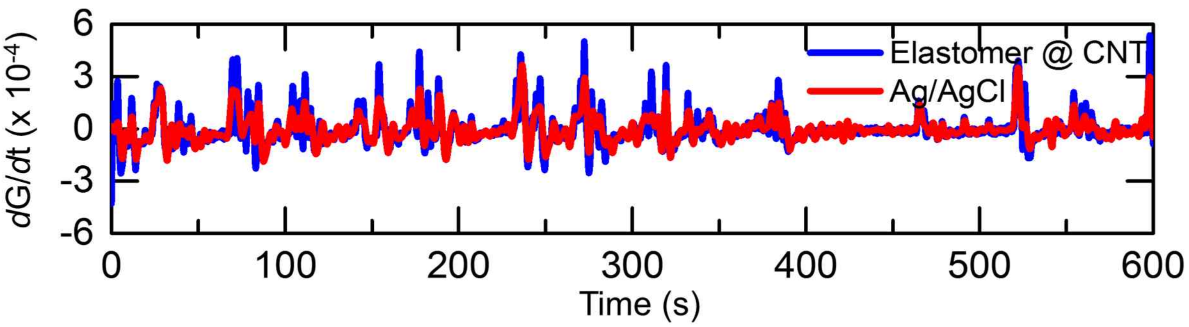

Supplementary Figure 12. Time-differential intensity of EDA signals detected by the two different sensors.

Supplementary Information 1. Analysis of electrical resistance of the SWCNT network-coated porous 3D network

To understand the change of the electrical resistance of the SWCNT network-coated elastomeric porous 3D network structure with respect to externally-applied compressive strain (ε), we constructed a simple mathematical model for the relationship between gauge factor (GF) and the strain ε.

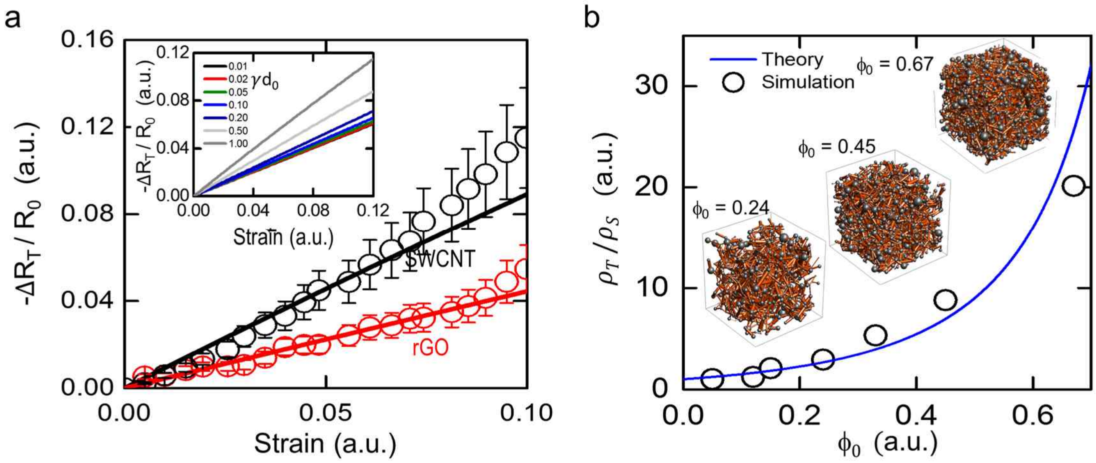

Supplementary Figure 13. a A relationship of $-\Delta R_T/R_0$ and ε with different conducting fillers. Inset plot is for the relationship between $-\Delta R_T/R_0$ and ε with different tunneling resistance parameters γd_0. **b** Comparison of theoretical calculation and simulation results for the relationship between ρ_T / ρ_S and ϕ_0. Inset illustrations are for the simulated network structures with different values of ϕ_0.

First, it is advantageous to use SWCNTs instead of rGO flakes. For instance, as shown in **Supplementary Fig. 13a**, given the same injected amount of rGO flakes and SWCNTs into the porous silicone elastomer medium (i.e., PDMS:Ecoflex® = 1:1 with the initial porosity (ϕ_0) of 0.607), the SWCNT network exhibited higher GF (i.e., a factor by 2.4) than that of the rGO network. The intrinsic tunneling resistance, R_i, can be written as[49, 51]

$$R_i = \frac{1}{n}\left(\frac{8\pi h d_0}{3\gamma A^2 e^2}\right)\exp\left(\gamma d_0\right) = \frac{L}{A^2}\rho_i,\ \gamma \equiv \frac{4\pi\left(2m\varphi\right)^{1/2}}{h}, \qquad (S1)$$

where n is the density of inter-flake conducting pathways which is equal to N/L (N and L are the

number of the conducting pathways and the average path length, respectively), h is the Plank's constant, d_0 is the tunneling distance before applying external stress, A^2 is the effective cross-sectional area among neighboring fillers, e is the electron charge, ρ_i is the intrinsic tunneling resistivity, m is the electron mass, and φ is the potential barrier. Typically, the tunneling resistivity is sufficiently greater than the intrinsic molecular resistivity of a single homogeneous conductive filler, and therefore, it is reasonable to consider R_i as an intrinsic resistance of the percolation network of the conductive fillers. When the conductive fillers uniformly coated on the surface of the porous elastic medium, they can work as a Kirchhoff resistance network, which fully functions as a closed circuit if the surface density of fillers is greater than a percolation threshold or the critical surface density.

Next, we modeled the effects of porosity on the gauge factor of the percolation network. For simplicity, we approximated the porous medium as an ideal 3D open-cellular (OCS) and linear elastic structure with characteristic cellular dimensions (i.e., cellular pillar thickness of t and length of l). Considering a fact that the percolation network of SWCNTs has sufficiently higher conductivity than the elastic materials composing the porous network (i.e., PDMS and Ecoflex®), we can obtain the effective resistivity of the SWCNTs network-coated pillar in the porous materials, ρ_S, such that $\rho_S \approx \rho_i t / 2h_f$, where h_f denotes the thickness of the thin film composed of the SWCNTs network coated on the surface of the pillar of the porous structure. For this relationship, we assumed that $t \gg h_f$. For a linear elastic OCS, it is well known that the effective conductivity of the entire porous structures, σ_T, is a function of geometric characters of the structure such that $\sigma_T / \sigma_S \propto (t/l)^2$ [52], where σ_S is the effective conductivity of the porous materials. Considering a porosity of the 3D OCS, ϕ, which has a relationship of $\phi \approx \frac{1-3(t/l)}{1+3(t/l)}$, where we assumed that $l \gg t$, we can obtain an expression of the effective resistivity of the entire porous structure (i.e., the 3D OCS with the inter-connected pillars coated with the SWCNT network), ρ_T, relative to the intrinsic resistivity ρ_S, such that $\frac{\sigma_S}{\sigma_T} = \frac{\rho_T}{\rho_S} = \left(\frac{1+\phi}{1-\phi}\right)^2$. We checked the dependence of ρ_T / ρ_S on ϕ using different simulated porous networks (modeled as a network composed of randomly-connected sticks-and-balls) with different values of ϕ with a simple resistor network model satisfying the Kirchhoff's current law[45, 53], we found that the

approximated expression for ρ_T/ρ_S matches well with the simulated results (**Supplementary Fig. 13b**).

In addition, it is also well known that the porosity is also a function of the external strain[54], which can be expressed as

$$\partial\phi = -f(\nu)\phi\partial\varepsilon, f(\nu) = \frac{1+\nu(\varepsilon)}{3[1-\nu(\varepsilon)]}, \quad (S2)$$

where $\nu(\varepsilon)$ denotes the Poisson's ratio of the porous structure which is dependent of the strain too. For the case of the OCS with overlapping spherical pores, a numerical study suggested that $\nu(\varepsilon)$ is a linear function of the porosity such that[55]

$$\nu(\varepsilon) = \nu_S + \frac{\phi(\varepsilon)}{\phi_0}(\nu_0 - \nu_S), \quad (S3)$$

where ν_0 and ν_S are Poisson's ratios of a porous structure with porosity of ϕ_0 with no application of the external strain and the elastic medium itself, respectively. Eqs.(S2) and (S3) can be solved using a numerical method, and we found that $\nu(\varepsilon)$ can also be expressed with a simple linear form such as $\nu(\varepsilon) = \nu_0 + \alpha\varepsilon$, given relatively small compressive strain (i.e., $\varepsilon < 0.45$). Using this approximated dependence of $\nu(\varepsilon)$, we can calculate a functional dependence of $\phi(\varepsilon)$ on ε such that

$$\phi(\varepsilon) \approx \phi_0 \left(\frac{1-\nu_0-\alpha\varepsilon}{1-\nu_0}\right)^{\frac{2}{3\alpha}} \exp[\varepsilon/3]. \quad (S4)$$

Further simplification of the form of $\phi(\varepsilon)$ is also possible using eqs.(S2) and (S4) as follows:

$$\phi(\varepsilon) \approx \phi_0 \exp\left[-\left(\frac{1+\nu_0+\alpha\varepsilon}{1-\nu_0-\alpha\varepsilon}\right)\frac{\varepsilon}{3}\right]. \quad (S5)$$

We observed that the effective Poisson's ratio of the entire OCS structure, $\nu(\varepsilon)$, also exhibits a nearly linear dependence on ε. It was also found that the slope describing the linearity, α, is also nearly linearly proportional to ν_S. This dependence of α on ν_S indicates that the more isotropic the elastic medium, the

stronger the linear dependence of ν on ε.

Using eqs.(S1) and (S5) in conjunction with other relationships found above, we can express the total resistance of the entire porous structure composed of inter-connected pillars which are surface-coated by the SWCNT network, R_T, as a function of ε as follows

$$R_T(\varepsilon)=\frac{t}{2n(\varepsilon)h_f}\left(\frac{8\pi hd(\varepsilon)}{3\gamma A^2e^2}\right)\exp(\gamma d(\varepsilon))\left(\frac{1+\phi_0\exp(-f(\nu)\varepsilon)}{1-\phi_0\exp(-f(\nu)\varepsilon)}\right)^2, \qquad (S6)$$

where $d(\varepsilon)$ can be assumed to follow a linear elastic behavior such as $d(\varepsilon)\approx d_0(1-\varepsilon)$[49, 51].

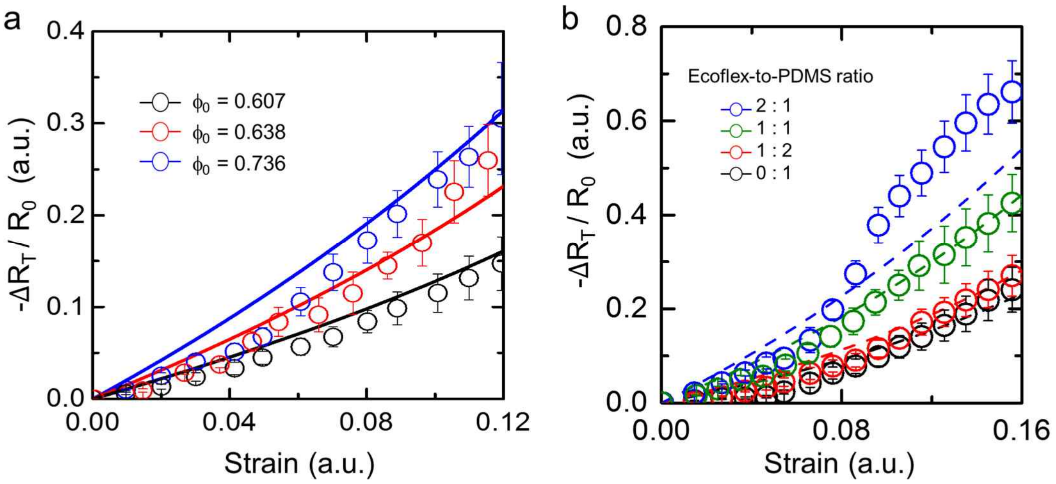

Supplementary Figure 14. a A relationship between $-\Delta R_T/R_0$ and ε with different porosities of 3D OCS fitted with a theoretical model (colored solid curves). **b** A relationship between $-\Delta R_T/R_0$ and ε with different elastic compositions of 3D OCS fitted with a theoretical model (colored dash curves).

Using eq.(S6), it is possible to calculate a ratio between R_T and the initial total resistance of the entire porous structure (prior to the application of compressive strain), R_{T0}, as follows:

$$\frac{R_T(\varepsilon)}{R_{T0}}=\frac{n_0(1-\varepsilon)}{n(\varepsilon)}\left(\frac{1-\phi_0}{1+\phi_0}\right)^2\exp(-\gamma d_0\varepsilon)\left[\frac{1+\phi_0\exp[-f(\nu)\varepsilon]}{1-\phi_0\exp[-f(\nu)\varepsilon]}\right]^2. \qquad (S7)$$

Using the simple mathematical model, we found that the change in $-\Delta R_T/R_0$ as a function of ε is well

described with common fitting parameters (i.e., $\alpha = 2.15$, $\nu_0 = 0.274$, and $\gamma d_0 = 2.11$), and the gradient of $-\Delta R_T / R_0$ with respect to ε is prominent for the case with the higher porosity (i.e., $\phi_0 = 0.736$) as shown in **Supplementary Fig. 14a**. It is also notable that the simple mathematical model can describe the difference of GF of the SWCNT network and the rGO-network with a single parameter, γd_0, which are responsible for the physical properties of the conducting fillers (i.e., $\gamma d_0 = 2.11$ for the SWCNT network and $\gamma d_0 = 0.501$ for the rGO network, respectively) with fixing other parameters concerning the physical properties of the elastic medium (i.e., $\alpha = 2.15$, $\nu_0 = 0.274$) as fitted in **Supplementary Fig. 13a**.

Supplementary Information 2. Effects of the elastic medium on GF

From eq.(S7), we found that GF increases with increasing the value of $f(\nu)$, which monotonically increases with increasing ν_0. It is reasonable to assume that ν_0 is proportional to ν_S for isotropic elastic medium[56, 57]. For an ideal soft rubber such as Ecoflex®, ν_S would be nearly equal to a value for the perfectly elastic material such as 0.5, while a hard rubber such as PDMS that is associated with cross-linking agent inside exhibits smaller value of ν_S (i.e., ~0.47)[58]. In our experiments, we observed that the porous structure composed of silicone rubber mixture with the higher fraction of Ecoflex® exhibited higher GF value. Referring to eq.(S7), the increase in the GF values can be partially attributed to the increases in ν_S, and in ν_0, subsequently. Another factor concerning medium properties affecting GF can be found in the surface properties. We observed that the SWCNTs-solution forms coffee-ring-like morphology when coated on the Ecoflex®-rich silicone rubber porous medium, while it forms island-like morphology when coated on the PDMS-rich silicone rubber porous medium. This difference in the morphology is due mainly to the interface energy between the SWCNTs-dispersed acetone and the silicone rubber as a function of the mixing ratio of Ecoflex®. Indeed, as shown in **Supplementary Fig. 5a**, the contact angle of SWCNTs-dispersed acetone on the surface of the elastic media decreases with increasing the mixing ratio of Ecoflex® in the PDMS rubber. Therefore, the volume occupied by a single SWCNT in the coated layer on the rubber surface would be greater in the case of the Ecoflex®-rich rubber than in the case of the PDMS-rich rubber. Then the average inter-distance among the SWCNTs fillers on the Ecoflex®-rich rubber surface would be greater than that on the PDMS-rich rubber surface. This difference can result in the greater tunneling distance for the SWCNTs on the surface of Ecoflex®-rich rubber than on the surface of PDMS-rich rubber. Subsequently, this can contribute to the increase in GF in the case of Ecoflex®-rich rubber. Considering a fact that a 3D OCS composed of a pure Ecoflex® rubber easily loses morphological stability, we determined the mixing ratio of Ecoflex®:PDMS as 2:1. The elastic modulus also decreased with increasing the Ecoflex® fraction as shown in **Supplementary Fig. 5a**. Therefore, Ecoflex®-rich elastic media can serve as a more flexible strain sensor.

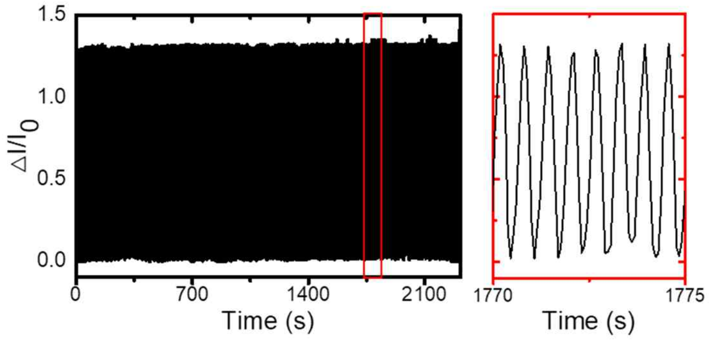

Supplementary Figure 15. Cyclic test of the active sensor during 3000 bending cycles under 25% strain. $\Delta I/I_0$ at 25% strain exhibited 0.8% standard error.

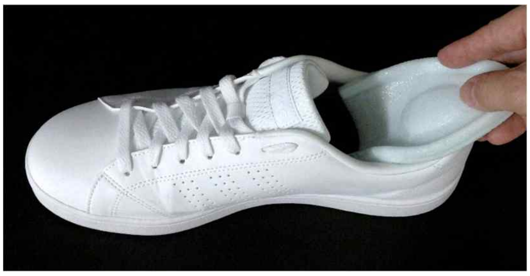

Supplementary Figure 16. A photograph of the insole-type wearable 3D-printed conductive patch-based sensor.

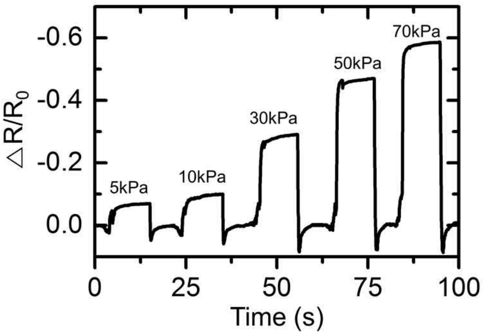

Supplementary Figure 17. Responses of the 3D-printed conductive patch-based sensor to the different levels of the pressure.

Supplementary Information 3. Analysis of signal-to-noise ratio of EMG data

For a time-dependent signal data $S(t)$, which is a set of N discrete signal points ($S(t)=\{s(t_i)\}$, where $i=1,2,\cdots,N$), the residual noise, $N(t)$, can be expressed as

$$N(t)=S(t)-S_d(t), \qquad (S8)$$

where $S_d(t)$ denotes the denoised time-dependent signal. Using $N(t)$, the signal-to-noise ratio (SNR) can be expressed as

$$SNR=10\log_{10}\left(\frac{\left\langle S(t)^2\right\rangle}{\left\langle N(t)^2\right\rangle}\right),\ \left\langle f(t)^2\right\rangle\equiv\frac{1}{N}\sum_{i=1}^{N}s(t_i)^2. \qquad (S9)$$

To compare SNR difference between two time-dependent signal data, $S_{stim}(t)$ and $S_{rest}(t)$, where the subscripts $stim$ and $rest$ denote periods under stimulation and of rest, respectively, we can calculate the relative SNR which can be expressed as

$$SNR_{rel}=SNR_{stim}-SNR_{rest}. \qquad (S10)$$

Using SNR_{rel}, we can quantitatively compare the deterioration dynamics of the neural signal transduction affected by degradation of the flexible electrode. Detailed procedure for the calculation of SNR is provided in Table S1.

Supplementary Table 1. An algorithm to calculate SNR of EMG signal data

Step	Description
1	Import raw data
2	Denoise the raw data using wavelet transform
3	Decompose the denoised signal using wavelet decomposition with various time scales
4	Calculate variance of the decomposed signal with various time scales
5	Find maximum variance change (gradient) point to define different types of the signal
6	Separate two types of the signal based on the maximum variance change point
7	Calculate residual noise for each of the signal types (stim & rest)
8	Calculate signal-to-noise ratio (SNR) for each of the signal types
9	Calculate relative SNR ratio between the two signal types

Supplementary References

[1] M. Marschollek, M. Gietzelt, M. Schulze, M. Kohlmann, B. Song, K. H. Wolf, *Healthc. Inform. Res* **2012**, *18*, 97.
[2] J. Rogers, Z. A. Bao, T. W. Lee, *Accounts. Chem. Res* **2019**, *52*, 521.
[3] C. X. Zhu, A. Chortos, Y. Wang, R. Pfattner, T. Lei, A. C. Hinckley, I. Pochorovski, X. Z. Yan, J. W. F. To, J. Y. Oh, J. B. H. Tok, Z. A. Bao, B. Murmann, *Nature Electronics* **2018**, *1*, 183.
[4] B. C. Gross, J. L. Erkal, S. Y. Lockwood, C. P. Chen, D. M. Spence, *Anal. Chem* **2014**, *86*, 3240.
[5] S. H. Ko, J. Chung, N. Hotz, K. H. Nam, C. P. Grigoropoulos, *J. Micromech. Microeng* **2010**, *20*, 125010.
[6] C. Ladd, J. H. So, J. Muth, M. D. Dickey, *Adv. Mater* **2013**, *25*, 5081.
[7] D. Bak, *Assembly. Autom* **2003**, *23*, 340.
[8] S. Hwang, E. I. Reyes, K. S. Moon, R. C. Rumpf, N. S. Kim, *J. Electron. Mater* **2015**, *44*, 771.
[9] J. H. Martin, B. D. Yahata, J. M. Hundley, J. A. Mayer, T. A. Schaedler, T. M. Pollock, *Nature* **2017**, *549*, 365.
[10] E. Vorndran, C. Moseke, U. Gbureck, *MRS. Bulletin* **2015**, *40*, 127.
[11] P. H. Warnke, H. Seitz, F. Warnke, S. T. Becker, S. Sivananthan, E. Sherry, Q. Liu, J. Wiltfang, T. Douglas, *J. Biomed. Mater. Res. B. Appl. Biomater* **2010**, *93*, 212.
[12] C. Minas, D. Carnelli, E. Tervoort, A. R. Studart, *Adv. Mater* **2016**, *28*, 9993.
[13] M. Mott, J. H. Song, J. R. G. Evans, *J. Am. Ceram. Soc* **1999**, *82*, 1653.
[14] U. Scheithauer, E. Schwarzer, H. J. Richter, T. Moritz, *Int. J. Appl. Ceram. Tec* **2015**, *12*, 26.

[15] E. Vorndran, M. Klarner, U. Klammert, L. M. Grover, S. Patel, J. E. Barralet, U. Gbureck, *Adv. Eng. Mater.* **2008**, *10*, B67.
[16] F. Castles, D. Isakov, A. Lui, Q. Lei, C. E. J. Dancer, Y. Wang, J. M. Janurudin, S. C. Speller, C. R. M. Grovenor, P. S. Grant, *Sci. Rep* **2016**, *6*, 22714.
[17] D. V. Isakov, Q. Lei, F. Castles, C. J. Stevens, C. R. M. Grovenor, P. S. Grant, *Mater. Des* **2016**, *93*, 423.
[18] S. C. Ligon, R. Liska, J. Stampfl, M. Gurr, R. Mulhaupt, *Chem. Rev* **2017**, *117*, 10212.
[19] X. Wang, M. Jiang, Z. W. Zhou, J. H. Gou, D. Hui, *Compos. Part. B-Eng* **2017**, *110*, 442.
[20] S. Bose, S. Vahabzadeh, A. Bandyopadhyay, *Mater. Today* **2013**, *16*, 496.
[21] A. El Sabbagh, M. F. Eleid, M. Al-Hijji, N. S. Anavekar, D. R. Holmes, V. T. Nkomo, G. S. Oderich, S. D. Cassivi, S. M. Said, C. S. Rihal, J. M. Matsumoto, T. A. Foley, *Curr. Cardiol. Rep* **2018**, *20*, 47.
[22] A. A. Giannopoulos, D. Mitsouras, S. J. Yoo, P. P. Liu, Y. Chatzizisis, F. J. Rybicki, *Nat. Rev. Cardiol* **2016**, *13*, 701.
[23] J. Gopinathan, I. Noh, *Biomater. Res* **2018**, *22*, 11.
[24] S. V. Murphy, A. Atala, *Nat. Biotechnol* **2014**, *32*, 773.
[25] C. L. Ventola, *Pharm. Ther* **2014**, *39*, 704.
[26] U. S. F. a. D. Administration, in *21*, Vol. 809 (Ed: U. S. F. a. D. Administration), 2019.
[27] R. P. Aquino, S. Barile, A. Grasso, M. Saviano, *Futures* **2018**, *103*, 35.
[28] Y. E. Choonara, L. C. du Toit, P. Kumar, P. P. D. Kondiah, V. Pillay, *Expert. Rev. Pharmacoecon. Outcomes. Res* **2016**, *16*, 23.
[29] M. Swan, *Int. J. Environ. Res. Public Health* **2009**, *6*, 492.
[30] G. D. Ltd, *Materials Data Book*, Cambridge University Engineering Department Granta Design Ltd **2003**.
[31] MatWeb, Technical Data Sheet PVA, http://www.matweb.com/search/DataSheet.aspx?MatGUID=ab96a4c0655c4018a8785ac4031b9278&ckck=1, accessed: 19th June, 2019.
[32] Q. Y. Soundararajah, B. S. B. Karunaratne, R. M. G. Rajapakse, *J. Compos. Mater* **2010**, *44*, 303.
[33] R. Melnikova, A. Ehrmann, K. Finsterbusch, *IOP. Conf. Ser. Mater. Sci. Eng* **2014**, *62*, 012018.
[34] Y. H. Zhang, F. Zhang, Z. Yan, Q. Ma, X. L. Li, Y. G. Huang, J. A. Rogers, *Nat. Rev. Mater* **2017**, *2*, 17019.
[35] A. K. Grosskopf, R. L. Truby, H. Kim, A. Perazzo, J. A. Lewis, H. A. Stone, *ACS. Appl. Mater. Inter* **2018**, *10*, 23353.
[36] K. Hajash, B. Sparrman, C. Guberan, J. Laucks, S. Tibbits, *3D Print. Addit. Manuf* **2017**, *4*, 123.
[37] S. Roh, D. P. Parekh, B. Bharti, S. D. Stoyanov, O. D. Velev, *Adv. Mater* **2017**, *29*, 1701554.
[38] A. D. Valentine, T. A. Busbee, J. W. Boley, J. R. Raney, A. Chortos, A. Kotikian, J. D. Berrigan, M. F. Durstock, J. A. Lewis, *Adv. Mater* **2017**, *29*, 1703817.
[39] T. J. Wallin, J. Pikul, R. F. Shepherd, *Nat. Rev. Mater* **2018**, *3*, 84.
[40] Y. Zhou, M. Layani, S. C. Wang, P. Hu, Y. J. Ke, S. Magdassi, Y. Long, *Adv. Funct. Mater* **2018**, *28*, 1705365.
[41] M. Kucewicz, P. Baranowski, J. Malachowski, A. Poplawski, P. Platek, *Mater. Des* **2018**, *142*, 177.
[42] D. Snelling, Q. Li, N. Meisel, C. B. Williams, R. C. Batra, A. P. Druschitz, *Adv. Eng. Mater.* **2015**, *17*, 923.
[43] H. Horacek, *J. Cell. Plast* **2016**, *52*, 189.
[44] A. M. Sakharov, V. G. Pimenov, *Vysokomol. Soedin* **1996**, *38*, 1729.
[45] X. J. Ni, C. Hui, N. H. Su, W. Jiang, F. Liu, *Nanotechnology* **2018**, *29*, 075401.
[46] I. Balberg, N. Binenbaum, C. H. Anderson, *Phys. Rev. Lett* **1983**, *51*, 1605.

[47] J. A. Quintanilla, R. M. Ziff, *Phys. Rev. E* **2007**, *76*, 051115.
[48] M. Teplan, *Meas. Sci. Rev* **2002**, *2*, 1.
[49] W. S. Bao, S. A. Meguid, Z. H. Zhu, G. J. Weng, *J. Appl. Phys* **2012**, *111*, 093726.
[50] H. Seo, S. Ahn, J. Kim, Y. A. Lee, K. H. Chung, K. J. Jeon, *Sci. Rep* **2014**, *4*, 5642.
[51] J. C. Li, C. H. Weng, F. C. Tsai, W. P. Shih, P. Z. Chang, *Appl. Phys. Lett* **2016**, *108*, 013108.
[52] K. P. Dharmasena, H. N. G. Wadley, *J. Mater. Res* **2002**, *17*, 625.
[53] B. J. Last, D. J. Thouless, *Phys. Rev. Lett* **1971**, *27*, 1719.
[54] R. W. Zimmerman, *Transport. Porous. Med* **2017**, *119*, 481.
[55] S. J. Sedler, T. R. Chase, J. H. Davidson, *J. Eng. Mater-T. ASME* **2017**, *139*, 011011.
[56] M. Arnold, A. R. Boccaccini, G. Ondracek, *J. Mater. Sci* **1996**, *31*, 1643.
[57] A. P. Roberts, E. J. Garboczi, *J. Am. Ceram. Soc* **2000**, *83*, 3041.
[58] S. Dogru, B. Aksoy, H. Bayraktar, B. E. Alaca, *Polym. Test* **2018**, *69*, 375.
